# Developing a prospective rapid-learning methodology to evaluate the survival impact of changing radiotherapy practice to include a new heart dose limit for patients with lung cancer in a UK specialist cancer centre (RAPID-RT): a protocol

**DOI:** 10.1136/bmjopen-2025-105519

**Published:** 2025-08-27

**Authors:** Isabella Fornacon-Wood, Rebecca Holley, Harry Crawford, Kathryn Banfill, Tom Marchant, Catharine Morgan, Hannah Turner-Uaandja, Abigail Walker, Evangelos Kontopantelis, Tjeerd van Staa, Sarah Devaney, Soren Holm, Gareth Price, Corinne Faivre-Finn

**Affiliations:** 1 Radiotherapy Related Research, Division of Cancer Sciences, Faculty of Biology, Medicine and Health, The University of Manchester, Manchester, UK; 2 Christie NHS Foundation Trust, Manchester, UK; 3 Division of Informatics, Imaging and Data Sciences, The University of Manchester, Manchester, UK; 4 Vocal, Research and Innovation Division, Manchester University NHS Foundation Trust, Manchester, UK; 5 NIHR School for Primary Care Research, The University of Manchester, Manchester, UK; 6 Centre for Health Informatics and Health Data Research UK North, Division of Informatics, Imaging and Data Science, School of Health Sciences, The University of Manchester, Manchester, UK; 7 Centre for Social Ethics and Policy, Division of Law, School of Social Sciences, The Faculty of Humanities, The University of Manchester, Manchester, UK

**Keywords:** ONCOLOGY, RADIOTHERAPY, STATISTICS & RESEARCH METHODS

## Abstract

**Abstract:**

**Introduction:**

The RAPID-RT study is part of a large-scale research programme investigating the use of routinely collected real-world patient data to rapidly and prospectively evaluate and optimise the impact of changes in radiotherapy practice on clinical outcome, an approach often referred to as ‘rapid learning’. As a proof of concept, a prospective, observational clinical study using realworld data is embedded within the programme. This study implements a new dose limit to a defined region of the heart in patients with stage I–III lung cancer treated with curative-intent radiotherapy at The Christie NHS Foundation Trust. The RAPID-RT study includes both methodological and clinical objectives. Its primary aim is to assess the feasibility and clinical acceptability of using rapid learning with real-world data to evaluate outcomes following modifications to standard-of-care radiotherapy protocols. This work has the potential to establish rapid learning as a robust, evidence-based approach for the continuous optimisation of radiotherapy workflows.

**Methods and analysis:**

RAPID-RT is a series of prospective single-arm observational studies with historic controls that uses only real-world data. A clinical decision was made to implement a dose limit to a specific region of the heart in all patients with stage I–III non-small cell lung cancer treated with curative-intent radiotherapy. The research focuses on using real-world data, the information collected as a part of patients’ routine care, to evaluate the impact of this change on overall survival and treatment-related toxicities. The study employs broad inclusion criteria, and data are extracted directly from the electronic patient record. Patients are provided with clear patient information materials and consent for data use via an informed opt-out process. Outcomes for patients treated before and after the introduction of the dose limit are compared using a Bayesian analytical framework to allow sequential updating of results as patients are recruited to the study. Evidence of clinical impact will guide the clinical team in determining whether refinements to the heart dose limit are necessary. These changes will, in turn, be evaluated in subsequent rapid-learning cycles. The RAPID-RT study aims to complete at least two iterative learning cycles to support the continuous optimisation of radiotherapy protocols.

**Ethics and dissemination:**

The study has received ethical approval (REC reference 22/NW/0390) from the North West Haydock Research Ethics Committee, is sponsored by The Christie NHS Foundation Trust and is funded by the UK National Institute for Health and Care Research. The programme management group is supported by an independent programme steering committee, an independent statistical review panel, a clinical management team and patient advisory group. Findings from the RAPID-RT study will be shared widely through conferences, focus groups and a stakeholder event, including a public ‘People’s Forum’ to co-create guiding principles for trusted rapid learning in radiotherapy. In parallel, interviews with participants, professionals and regulators will inform consensus and the development of practical, ethical and legal guidelines to support the adoption of rapid learning across NHS radiotherapy centres.

**Trial registeration number:**

ISRCTN17129364.

**STRENGTHS AND LIMITATIONS OF THIS STUDY**

- Use of a historical control design enables evaluation of treatment changes made in the routine setting where randomisation is often not feasible and changes in care are driven by clinical decisions.
- Broad inclusion criteria and an informed opt-out consent process balance patient choice with improved study generalisability and inclusivity.
- Use of routinely collected clinical data supports rapid patient recruitment but requires quality assurance to enhance the accuracy of structured electronic health record data.
- Bayesian methods support adaptive recruitment, allowing dynamic evaluation of evolving treatments in successive learning cycles.
- As a low-cost, pragmatic approach, the study is scalable, though residual confounding remains a limitation due to the non-randomised design.

## Introduction

 Radiotherapy plays a key role in the treatment of lung cancer, with over 60% of patients receiving it as part of their care.^1 2^ Standard of care radiotherapy protocols are frequently updated to optimise treatment outcomes. While some changes are informed by evidence from randomised controlled trials (RCTs), many are also driven by findings from observational studies or prompted by the integration of new technologies into the treatment pathway.

The clinical impact of such changes in practice can be challenging to evaluate in formal RCTs as they are not well suited in this context; for both technical (eg, lack of equipoise) and practical (resource, fit with routine practice) reasons.^3^ As a result, formal prospective assessment of the impact of such changes in the real world is lacking. Furthermore, many patient groups, such as the elderly, patients with multiple comorbidities, ethnic minorities and those who are socioeconomically deprived, are under-represented in clinical trial cohorts even when they fit inclusion criteria,^4 5^ raising issues of the generalisability of trial results to the patient populations seen in radiotherapy clinics and potentially compounding health inequities.

There is, therefore, an unmet need to develop methodologies to rapidly evaluate changes to clinical practice made in the routine setting to ensure they are beneficial to patients, or at the least not detrimental, and help drive improvements in cancer care. The rapid learning concept proposes evaluating changes to practice using real-world data, data collected as a part of routine care, to rapidly provide evidence of the impact of changes to practice on clinical and operational outcomes.^6^ As real-world data are collected for all patients as a part of routine care, the evidence generated should be applicable to the entire local patient population. NHS England recognises this unmet need and pledged to establish a Learning Healthcare System for radiotherapy in England in 2019.^7^ Despite interest in rapid learning and Learning Healthcare Systems, to date there is a lack of implementation clinically.^6^

A key example of a change to routine clinical practice is the introduction of a novel dose limit to a previously unrecognised organ at risk in patients with lung cancer treated with radiotherapy. Emerging evidence has demonstrated that higher radiation doses to the heart are associated with poorer survival and increased cardiac events in this population,^8 9^ with further studies identifying a specific region at the top of the heart that is associated with poorer outcomes.^10^,^13^ In response, the lung cancer team at The Christie NHS Foundation Trust made a clinical decision to introduce a new dose limit for this region, with the aim of improving overall survival. The RAPID-RT study is evaluating the use of rapid learning with real-world data to formally assess the impact of this change, although the decision to implement the dose limit was made independently of the study.

The RAPID-RT study is part of a large-scale research programme exploring the use of routinely collected real-world data to rapidly and prospectively evaluate and optimise the impact of changes in radiotherapy practice, an approach known as ‘rapid learning’. This prospective, observational study serves as a proof of concept within the wider programme.^14^ Other work packages address key challenges to implementing rapid learning in the routine clinical setting, including ethical acceptability, health economics, applicability to other changes in radiotherapy practice and organisational barriers.

## Methods and analysis

RAPID-RT is a non-interventional observational study with a historic control group using real-world data. All patients with stage I–III lung cancer treated with curative-intent radiotherapy (excluding stereotactic ablative body radiotherapy) at The Christie NHS Foundation Trust are enrolled into the RAPID-RT study with the option to opt out. Data are collected for a retrospective cohort of patients treated before the implementation of the dose limit to the top of the heart, and for a prospective cohort treated after its implementation in April 2023. The clinical data are collected as a part of routine care within The Christie’s electronic patient record using structured electronic forms filled in by the clinical team. Patients are followed as per standard-of-practice protocol. The study started in April 2023 and is due to complete in January 2027.

The RAPID-RT trial includes both methodological and clinical objectives, as described in table 1.

**Table 1:** Summary of the RAPID-RT study’s methodological aims and objectives, and those of the clinical study used to establish, demonstrate and evaluate these

	Methodology study	Clinical study
Aims	Development of the ‘rapid learning’ processes and methodology needed to evaluate changes to routine care made outside of research protocols as an embedded part of normal practice.	An exemplar change in routine care, the introduction of a new radiotherapy dose limit for a region of the heart, that is typically introduced without formal trial evaluation. Used to establish, demonstrate and evaluate the rapid-learning methodology.
Primary objective	Assess the feasibility and clinical acceptability of using rapid learning with real-world data to evaluate outcomes following modifications to standard-of-care radiotherapy protocols.	To assess the impact on overall survival at 1 year post-radiotherapy.
Secondary objectives	Establish observational study database. Assess the quality of the real-world data captured in study database. Develop and evaluate statistical framework to evaluate changes in standard of care treatments using real-world data. Investigate the practicalities of using informed opt-out consent in a rapid learning study.	To assess the impact on acute toxicities, specifically severe oesophagitis and pneumonitis between the start and 6 months post-radiotherapy (≥grade 3 according to the Common Terminology Criteria for Adverse Events V.5). To assess the impact on patient-reported outcomes symptoms (Common Terminology Criteria for Adverse Events V.5) and quality of life (EQ-5D).

EQ-5D, EuroQol-5D.

### Participant screening and selection

Patients are screened in the lung cancer clinics at The Christie NHS Foundation Trust by the direct care team. The study employs broad inclusion criteria. There are no exclusion criteria relating to patient comorbidities, age or performance status.

### Inclusion criteria

- Patients with stage I–III lung cancer.
- Patients treated with curative-intent radiotherapy.
- Patients with the capacity to consent to their radiotherapy treatment.

### Exclusion criteria

- Patients under 18 years of age.
- Patients treated with stereotactic ablative body radiotherapy.
- Patients who have opted out of the study.

### Informed opt-out consent

This study incorporates an informed opt-out consent model that was informed by a citizen jury involving patients and the public.^15^ Patients are informed that their de-identified, routinely collected data from their cancer care will be used for this study. A simplified 2-page participation information sheet that describes the study and how participants can opt out is provided to eligible patients by the clinical team at their first appointment (see online supplemental data). Patients also have the option to watch a video as an alternative (https://sites.manchester.ac.uk/rapid-rt/).

### Cardiac avoidance area and the introduction of dose limit

The introduction of a dose limit to the top of the heart (or cardiac avoidance area (CAA)) was a clinical decision made by the Christie Lung team.

Based on the association between excess dose to this region and reduced overall survival,^10^ and on considerations of cardiac physiology,^8^ an anatomical region known as the CAA was defined by a multidisciplinary team at The Christie NHS Foundation Trust, including a cardiologist, clinical oncologists and medical physicists. The CAA includes the right atrium, aortic valve root, coronary artery ostia and proximal right and left coronary arteries.^16^

To facilitate the implementation of a heart dose limit, a deep learning-based auto-contouring algorithm was developed and clinically validated for use during radiotherapy treatment planning, the first stage of which requires the tumour target and nearby healthy tissues to be defined in the planning CT scan. This enables consistent and rapid delineation of the CAA in the routine setting.^17^ The clinical care team remains responsible for reviewing and verifying this contour as part of standard quality assurance.

As part of this change, The Christie’s lung radiotherapy planning process was updated to include a maximum dose objective for the CAA of 19.5 Gy in 20 fractions (or equivalent biologically effective dose^18^). This threshold was based on the findings by McWilliam *et al*,^11^ which identified it as the dose threshold associated with the most significant difference in overall survival. In cases where the planning target volume (PTV) overlaps with or is close to the CAA, PTV coverage will be prioritised over CAA dose reduction. In such instances, the maximum dose limit will only be applied to the portion of the CAA located more than 1.5 cm from the PTV. Analysis of planned dose distributions demonstrated a significant reduction in CAA and whole heart doses following implementation of the limit, with no significant impact on doses to other thoracic organs at risk.^19^

### Outcome measures

The methodological and clinical outcome measures are described in table 1.

### Data collection

No additional data outside of that collected routinely at The Christie NHS Foundation Trust are collected for this study. Data on patients and tumour characteristics and treatment-related toxicities are collected routinely before, during and after radiotherapy in structured forms within the electronic patient records at The Christie. The structured forms are filled in by the clinical team in real time. Case report forms are not used.

**Figure 1:**
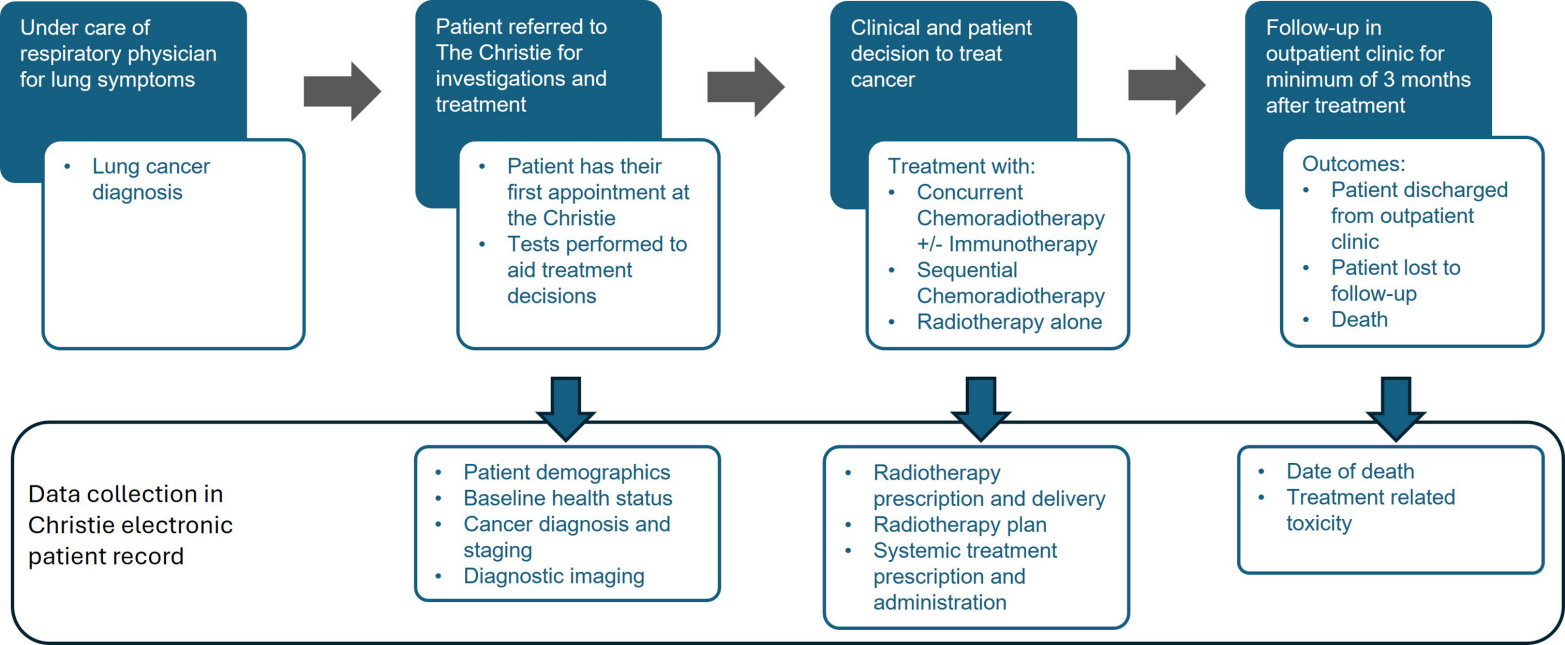
The treatment pathway for patients with lung cancer at The Christie NHS Foundation Trust showing data captured in the Christie electronic record at each stage of the process.

The treatment pathway for patients diagnosed with lung cancer is depicted in figure 1, with electronic forms completed at different stages along this pathway. At a patient’s first appointment, a baseline form is generated which records information about a patient, such as demographics, comorbidities, performance status, histology, tumour, node, metastases stage and treatment intent. Further electronic forms are generated at subsequent outpatient review appointments, containing both structured and unstructured data. The structured fields include performance status, disease status and information about the worst toxicity experienced by the patient since the previous appointment, according to Common Terminology Criteria for Adverse Events V.5. The unstructured fields contain free text summaries of the patient consultation.

For patients undergoing radiotherapy treatment, outpatient appointments typically take place pretreatment, 6 weeks after the end of treatment and subsequently at 3 months, 6 months and 12 months post-treatment. However, some patients may be discharged earlier or later depending on clinical decisions. Information about the treatments received is recorded electronically in the radiotherapy prescription system (Mosaiq) and systemic therapy prescribing system (iQemo).

Diagnostic and follow-up images are stored in the Greater Manchester Picture Archiving and Communication System. Individual radiotherapy plans are bespoke for each patient to treat the tumour while limiting dose to healthy tissues. Plans are created on CT scan images and after use are archived locally in DICOM-RT format.

The data items required for the RAPID-RT study are automatically extracted from The Christie electronic patient record, pseudo-anonymised on a dedicated system and then pushed into the RAPID-RT database for access by the study team.^20^ Images and radiotherapy plans are automatically retrieved from the Greater Manchester PACS and local archives and linked with a study ID to the pseudo-anonymised clinical data via the key-value table on the anonymisation system. The research team does not have access to the pseudo-anonymisation server, only the systems containing the already de-identified study data.

The RAPID-RT database includes only patients who did not opt out from data use for the study. All data are stored on a secure server in compliance with all Trust information governance and research policies.

### Historical control group

The historical control cohort will be extracted from an established research database that permits secure access to anonymised routinely collected data sources at The Christie NHS Foundation Trust (UK Research Ethics Committee ref. 21/NW/0347). This retrospective dataset comprises patients treated with curative-intent radiotherapy for stage I–III lung cancer between January 2021 and April 2023, prior to the implementation of the CAA dose limit. Patients included in the control group meet the same eligibility criteria as those in the prospective RAPID-RT cohort and have not opted out of data use under the UK’s National Data Opt-Out. This cohort is expected to include approximately 1000 patients, providing a robust comparator group for evaluating the impact of the new dose limit using Bayesian analytical methods.

### Data quality

The electronic record of all patients who receive the new radiotherapy standard-of-care described above is checked manually by a research practitioner, who is patient-facing and authorised to access clinical records. They retrospectively review clinical notes to ensure that any toxicity documented in unstructured text is also recorded in a structured form. Severe respiratory and oesophageal toxicities (≥grade 3) are flagged for independent review by two clinicians to accurately assess their relatedness to radiotherapy.

If toxicity events are identified in unstructured text without a corresponding structured record, a structured entry is created by generating a new follow-up form, with the date adjusted to reflect the timing of the original event. If no toxicity is found in unstructured data and there is no structured record (including confirmed absence of toxicity), a structured entry is added to confirm the absence of radiotherapy-related toxicity. In some cases, patients are contacted directly to clarify the presence and severity of a toxicity event, particularly when follow-up is no longer taking place at The Christie, as relevant data from other hospitals may not be incorporated into the patient’s electronic record in structured format.

This work will support an assessment of the quality of structured data in The Christie electronic record and help estimate the resources and processes required to deliver data of sufficient quality for use in similar real-world data studies.

### Statistics

As one of the key objectives of the RAPID-RT programme is to develop and implement a statistical framework to assess clinical impact using real-world radiotherapy data, the analysis plan is not fully defined a priori. The goal of the statistical framework is to incorporate data from patients immediately after treatment completion, with clinical outcomes continuously updated throughout follow-up.

Outcomes will be analysed by comparing cohorts before and after any change in care. In the clinical example we use here, the first analysis will include a retrospective cohort (cohort 1) treated before the introduction of the dose limit and a prospective cohort (cohort 2) treated after its implementation. A Bayesian parametric survival model will be used, enabling continuous real-time updating of effect estimates as data accrue.^21 22^ This approach supports flexible, adaptive analysis, for example, by allowing learning cycles to conclude early or be extended, depending on the observed effect size, and avoids the need for prespecified interim timepoints and adjustments for multiple comparisons. Survival outcomes will be analysed using a parametric survival model, which allows adjustment for confounding and imbalance between cohorts. The model will include key prognostic factors identified through feature selection or designated as clinically important by the care team. Initial Bayesian estimation of model coefficients will use uninformative priors, with later models incorporating sceptical and optimistic informative priors as appropriate. Missing data will be addressed using standard multiple imputation techniques.^23^

**Figure 2:**
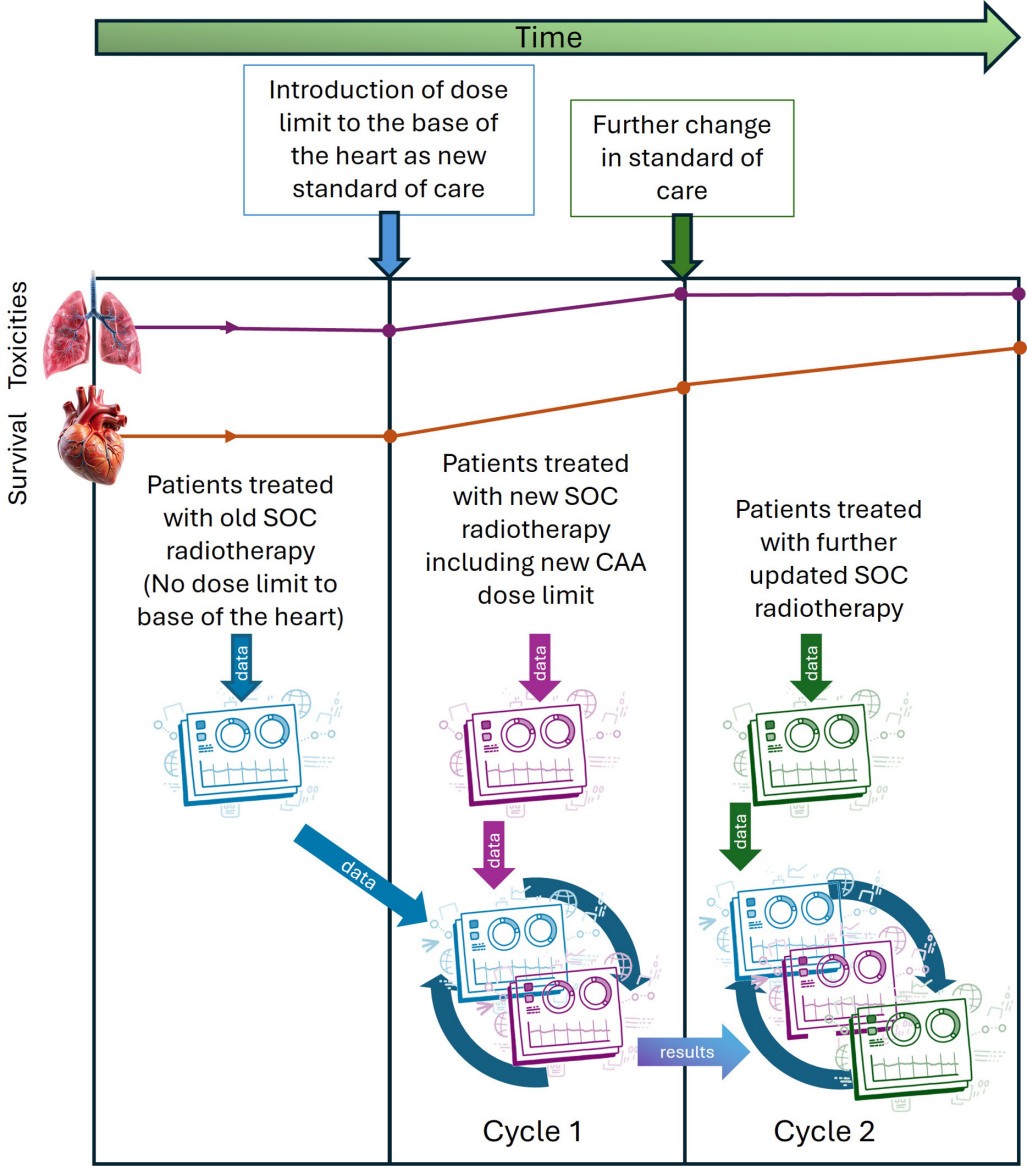
Potential rapid learning cycles. CAA, cardiac avoidance area. SOC, Standard of care.

Results will be reviewed regularly by The Christie lung cancer direct care team, who will determine whether further modifications to the heart-sparing technique are warranted. These decisions will be made independently by the clinical team and fall outside the scope of this research protocol. If any further changes are introduced, a new rapid-learning cycle will be initiated from the date of implementation. Outcomes from each subsequent cycle will be compared with those from preceding cohorts (see figure 2).

Based on the number of newly diagnosed patients seen at the Christie NHS Foundation Trust per month, approximately 40 per month are expected to be eligible for inclusion in RAPID-RT, equating to around 480 per year. Depending on the estimated effect size of the new dose limit on survival (estimated ~10% increase in survival at 1 year based on retrospective studies^11^), a sample of 750–1000 patients is anticipated to provide sufficient data to determine the probability of its impact with high confidence.

The study will end on completion of data collection and once the database has been locked. A statistical analysis plan will be developed and independently reviewed before study analysis. Both the methodological insights gained from the rapid-learning approach and the clinical outcomes from the exemplar evaluation will be published and shared with relevant stakeholders.

### Patient and public involvement and engagement

Public involvement and engagement is central to the RAPID-RT programme. In collaboration with Vocal, an organisation that connects people and health research,^24^ a structured patient and public involvement and engagement (PPIE) framework has been developed to ensure that patients, carers and the public contribute meaningfully to the design and conduct of the study.

Two patient co-applicants work in partnership with Vocal and co-chair a RAPID-RT Patient Advisory Group (PAG), who provide ongoing review and advice on all patient-facing aspects of the programme. Additional PPIE activities have included a 2-day citizens’ jury, convened to determine the most appropriate consent model for the use of anonymised patient data in RAPID-RT.^15^ The jury included 24 public or patient members, including individuals with lived experience of radiotherapy or cancer treatments. Jurors heard evidence from experts in study design, healthcare law and ethics, data governance and consent processes. 74% of jurors concluded that an informed opt-out consent model was preferable to a traditional written consent approach. Jurors also provided input on communication preferences, unanimously recommending a concise, written short information sheet, introduced by a clinician. These recommendations have been fully adopted in the design of the study’s consent process. Patients were also actively involved in the co-design of patient-facing focus groups exploring the acceptability and understanding of rapid learning. This process included the development of an animation designed to explain the concept of rapid learning in a patient-friendly and accessible format (https://sites.manchester.ac.uk/rapid-rt/).

### Patient and clinical experience of the opt-out approach to health data use in the RAPID-RT study

To evaluate the acceptability of the informed opt-out model used for data inclusion in RAPID-RT, a Study Within a Trial (SWAT) will be conducted. This SWAT will involve semi-structured qualitative interviews, with a purposive sample of up to 30 patients eligible for inclusion in RAPID-RT and up to 20 members of the clinical team responsible for introducing the Patient Information Sheet. The aim of these interviews is to gather evidence on patients’ and clinicians’ experiences of the opt-out approach, including its perceived acceptability, clarity and inclusiveness. Findings will inform the research team about the appropriateness of this consent model and help optimise future implementation of rapid-learning research.

## Ethics and dissemination

The study has received ethical approval (REC reference 22/NW/0390) from the UK’s North West Haydock Research Ethics Committee.

### Safety reporting

As an observational study, RAPID-RT does not involve investigational treatments or interventions. Clinical outcomes are reported to the direct care team to inform decisions about learning cycles, but are not submitted to regulatory authorities for safety reporting.

### Project monitoring

**Figure 3:**
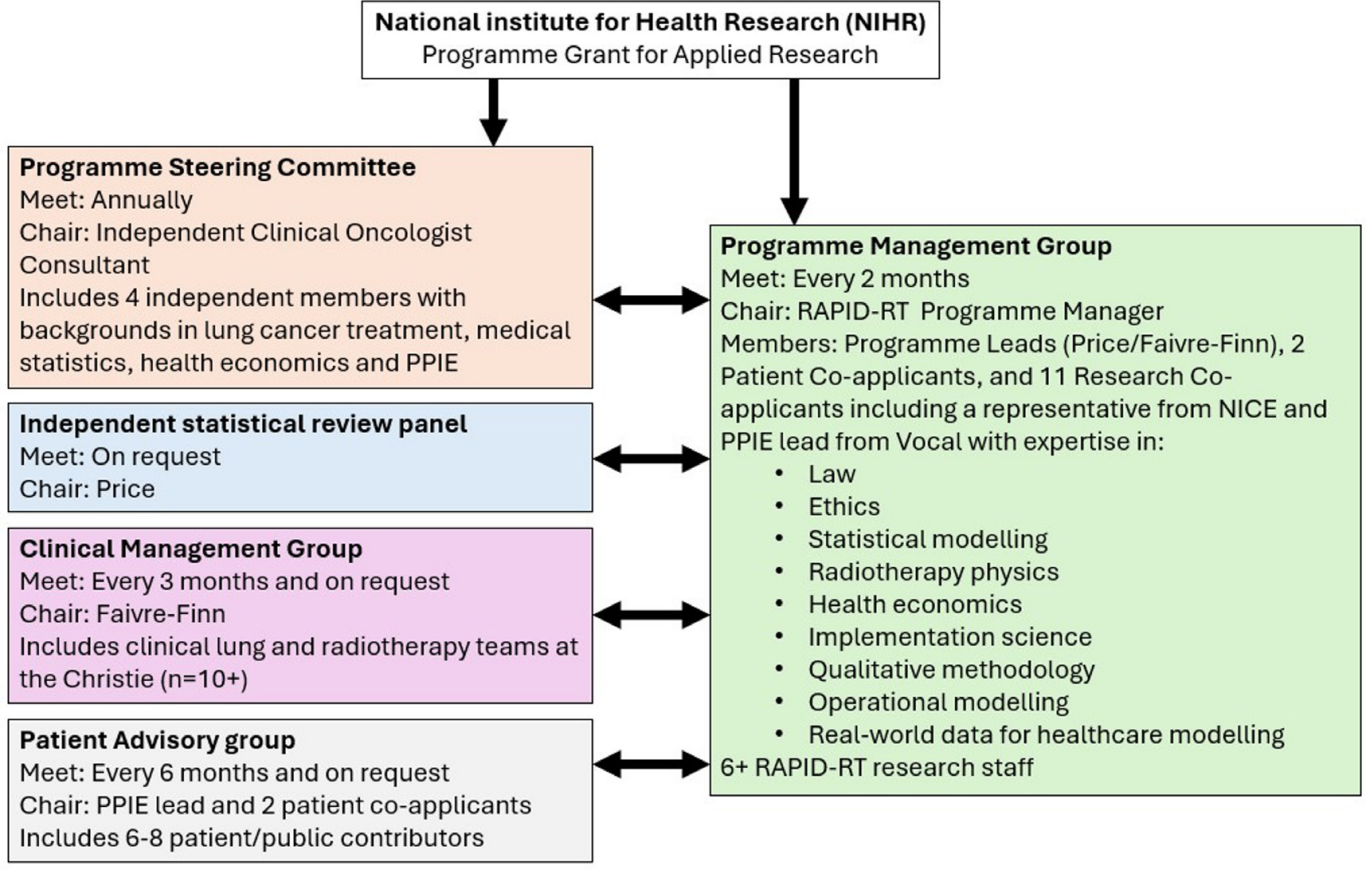
Overview of RAPID-RT management structure. PPIE, patient and public involvement and engagement. NICE, National Institute for Health and Care Excellence

Project oversight for RAPID-RT is structured across several governance bodies (see figure 3). A programme management group (PMG) comprising all research team members and programme co-applicants meets every 2 months to monitor progress, review deliverables and coordinate research activities. Clinical oversight is provided by a clinical management group consisting of clinicians involved in the radiotherapy care of patients with lung cancer. The group is responsible for all clinical decisions regarding the recruitment to the study and reviewing outcomes to inform potential changes to cycles of learning. An independent programme steering committee meets annually to assess progress against key milestones, report to the funder and provide strategic advice on necessary adaptations. A dedicated programme manager oversees the day-to-day operations of the study. An Independent Statistical Review Panel evaluates the statistical framework and analyses, offering expert recommendations to the PMG prior to application of findings to clinical practice.

The PAG, co-chaired by two patient co-applicants, meets biannually—or more frequently as needed—to review all patient-facing materials. PAG representatives also participate in the PMG to provide feedback on patient and public involvement activities and identify opportunities to strengthen patient engagement.

### Dissemination

Findings from both the main methodological and clinical aims of the RAPID-RT study will be published and publicised through regional, national and international conferences. To inform dissemination and future guidelines, focus groups and a large stakeholder event with patients and members of the public will be held. This will include a ‘People’s Forum: Co-Creating the Charter for Rapid Learning in Radiotherapy’, a collaboratively developed framework of guiding principles, designed not to provide rigid answers, but to reflect the conditions under which the public believes rapid learning can be acceptable and trusted.

In parallel, interviews and discussion groups with clinicians and healthcare professionals, regulators and other key stakeholders will support the development of a consensus on when and how rapid learning should be adopted in NHS radiotherapy centres and more broadly. Guidelines addressing the legal, ethical and practical aspects of rapid learning implementation will be published to support adoption in other centres.

## Supplementary material

10.1136/bmjopen-2025-105519 online supplemental file 1
