# Supplementary material for: Developing a prospective rapid-learning methodology to evaluate the survival impact of changing radiotherapy practice to include a new heart dose limit for patients with lung cancer in a UK specialist cancer centre (RAPID-RT): a protocol
Source: BMJ Open. 2025 Aug 27;15(8):e105519. doi: 10.1136/bmjopen-2025-105519 (PMC12410672; doi:10.1136/bmjopen-2025-105519)
Supplement: online supplemental file 1 [file bmjopen-15-8-s001.pdf]

RAPID-RT: a study using patients' routine data to improve care

Information for Patients

This is an opt-out study

This means data relating to your cancer treatment will be included in the study unless you tell us not to

Consultants often make small updates to the way cancer treatments are given at The Christie. These changes are made to improve the way patients are cared for. The changes are based on research results, and on information from other patients or other treatment centres.

RAPID-RT is a research study that will develop a new method to bring together and analyse **anonymised** data collected from the medical records of patients receiving radiotherapy for their lung cancer. This data will be used to develop a method to help us work out whether changes made to treatments in the clinic are improving patient care. We can include your data in this study because you have stage I, II, or III lung cancer and are receiving radiotherapy to treat it.

The more data we include, the more sure we can be that the results of RAPID-RT are right. **Letting us use your data will not change anything about your cancer or its treatment** or mean you will have any extra trips to hospital or tests.

What happens if you use my data in RAPID-RT?

- Nothing about your cancer treatment will change
- We will use only data from your medical records that relates to your cancer treatment
- All of your information will be stored safely, securely and confidentially
- It will have your name and other private information removed (this is also called 'anonymised' data) before it is analysed by the research team
- Your confidentiality will be protected and people who do not need to know who you are will not be able to identify you from the data used in this study. They will never be able to see your name or your contact details.
- At the end of the study, we will save some of the data, in case we need to check it or use it in future research.

How do I opt out?

- If you do **not** want your information to be used in this study, **you must tell us**
- This is called **opting out** and you can do it at any time without giving a reason
- It will **always** be your choice to opt out

If you decide you do not want your data to be used in the study, please tell a member of your Christie care team and we will make sure it is not used. You can also email the-christie.rapidrt.patients@nhs.net

What happens if I opt out?

Your cancer treatment will not change if you opt out.

If you opt out before the end of your course of radiotherapy, RAPID-RT will not use any of your data.

If you opt out later in your cancer pathway, no further data will be used. However, if some of your information has already been included in an analysis, this will be impossible to remove as it will be anonymous. This means we cannot trace it to you and so cannot remove it.

If you lose the capacity to opt out during the study, we will retain your data in the analysis.

You might have already chosen to opt out of health data collection via the NHS National data opt-out service. Data used for RAPID-RT will only include data collected at the Christie about your cancer treatment. Therefore, if you want to, you can still allow us to use this data in RAPID-RT without affecting your national opt-out status.

What benefits and risks are there to me of taking part in this study?

Nothing about your cancer treatment changes if you take part in this study. Therefore, there are no risks or benefits to you personally from letting us include your data. You would be helping to develop a method to help us see how changes in treatments are improving patient outcomes. Knowing this information may benefit patients with lung cancer in the future.

You can find out more about how we use your information:

- At www.hra.nhs.uk/information-about-patients
- By reading the following patient leaflet: http://www.hra.nhs.uk/patientdataandresearch
- By asking one of the research team
- By sending an email to the-christie.dpo@nhs.net

Who runs RAPID-RT?

RAPID-RT is run by The Christie NHS Foundation Trust and funded by the National Institute for Health Research. It has been approved by an independent NHS Research Ethics Committee, who will make sure that your safety and rights are respected, and by the hospital where you are being treated.

Concerned or want to know more?

If you have any concerns or questions about RAPID-RT, please contact Prof Corinne Faivre-Finn's secretary on 0161 446 8200.

If you would like to make a formal complaint, you can do this through the NHS Complaints Procedure. Details can be obtained from your Christie care team.

If you are interested to know more, visit our website:
https://sites.manchester.ac.uk/rapid-rt/

Updates on the progress and results of the study will also be available on our website.

This information sheet is available in audio form: https://vimeo.com/810128202/34777b21fd

There is also a patient information video describing the study:
https://vimeo.com/806008044/d64b2ff30c

If you would like to receive a copy of the study outcomes when available, please email:
the-christie.rapidrt.patients@nhs.net.

You will not get a copy of the study outcomes unless you email us.
