# Supplementary material for: Developing a prospective rapid-learning methodology to evaluate the survival impact of changing radiotherapy practice to include a new heart dose limit for patients with lung cancer in a UK specialist cancer centre (RAPID-RT): a protocol
Source: BMJ Open. 2025 Aug 27;15(8):e105519. doi: 10.1136/bmjopen-2025-105519 (PMC12410672; doi:10.1136/bmjopen-2025-105519)
Supplement: Supplementary file 2 [file bmjopen-2025-105519.reviewer_comments.pdf]

PEER REVIEW HISTORY

BMJ Open publishes all reviews undertaken for accepted manuscripts. Reviewers are asked to complete a checklist review form and are provided with free text boxes to elaborate on their assessment. These free text comments are reproduced below.

ARTICLE DETAILS

Title (Provisional)

Protocol: Developing a prospective rapid-learning methodology to evaluate the survival impact of changing radiotherapy practice to include a new heart dose limit for lung cancer patients in a UK specialist cancer centre (RAPID-RT)

Authors

Fornacon-Wood, Isabella; Holley, Rebecca; Crawford, Harry; Banfill, Kathryn; Marchant, Tom; Morgan, Catharine; Turner-Uaandja, Hannah; Walker, Abigail; Kontopantelis, Evangelos; van Staa, Tjeerd; Devaney, Sarah; Holm, Soren; Price, Gareth; Faivre-Finn, Corinne

---

This article was not externally reviewed at BMJ Open. Protocols that have been independently assessed prior to submission to BMJ Open are usually fast-tracked to publication on the grounds that further substantial changes will not be possible. This independent assessment will usually be external, independent review for both a substantial grant award from a non-commercial or government funder and ethics approval.
